# Supplementary material for: Extending the toolbox for RNA biology with SegModTeX: a polymerase-driven method for site-specific and segmental labeling of RNA
Source: Nat Commun. 2023 Dec 18;14:8422. doi: 10.1038/s41467-023-44254-3 (PMC10728113; doi:10.1038/s41467-023-44254-3)
Supplement: Supplementary file 1 — Supplementary Information [file 41467_2023_44254_MOESM1_ESM.pdf]

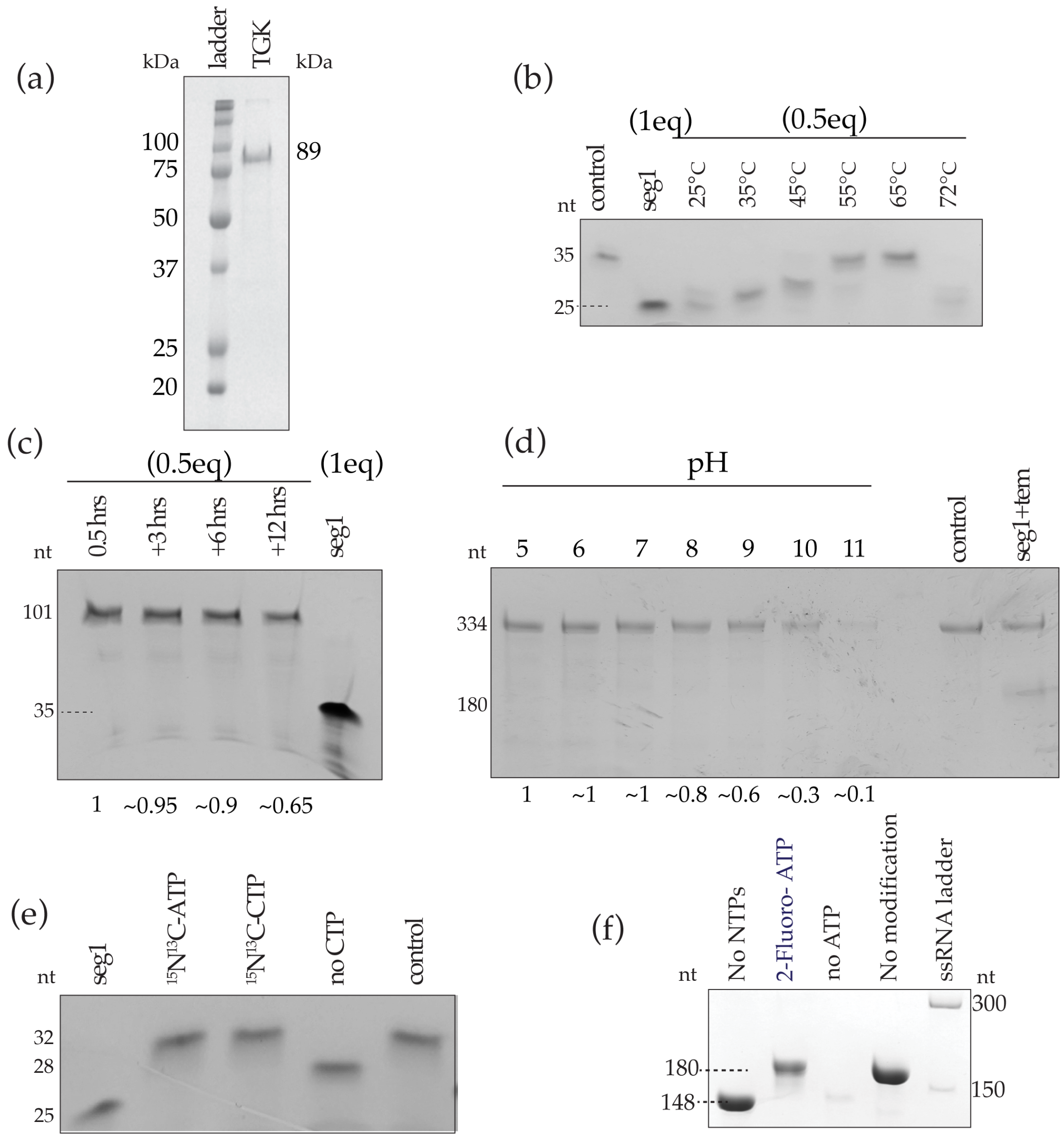

Supplementary Figure 1: Optimization of SegModTeX reaction conditions

(a) SDS-PAGE showing quality of TGK polymerase after purification. **(b)** Hourlong extension reactions at varying temperatures. No extension is visible at 72°C as it far exceeds the annealing temperature of 58°C. **(c)** Extended exposure of SegModTeX reaction at 72°C over the course of 12h showing the approximate yields (below gel) decreases over time. **(d)** Extension reactions at varying pHs indicate poor yields above pH 9. **(e)** Extensions using $^{15}N^{13}C$ labeled rNTPs (lanes 2 and 3) readily incorporate similar to unlabeled rNTPs (lane 5). **(f)** A 148-nt RNA seg-1 (lane 1) with CTP, UTP, GTP, and 2-19^{F}-2-^{13}C-ATP by a 30-nt segment encoding 5 A's (lane 2) extends to the expected length (lane 4). Lack of an ATP analogue stalls the reaction (lane 3).

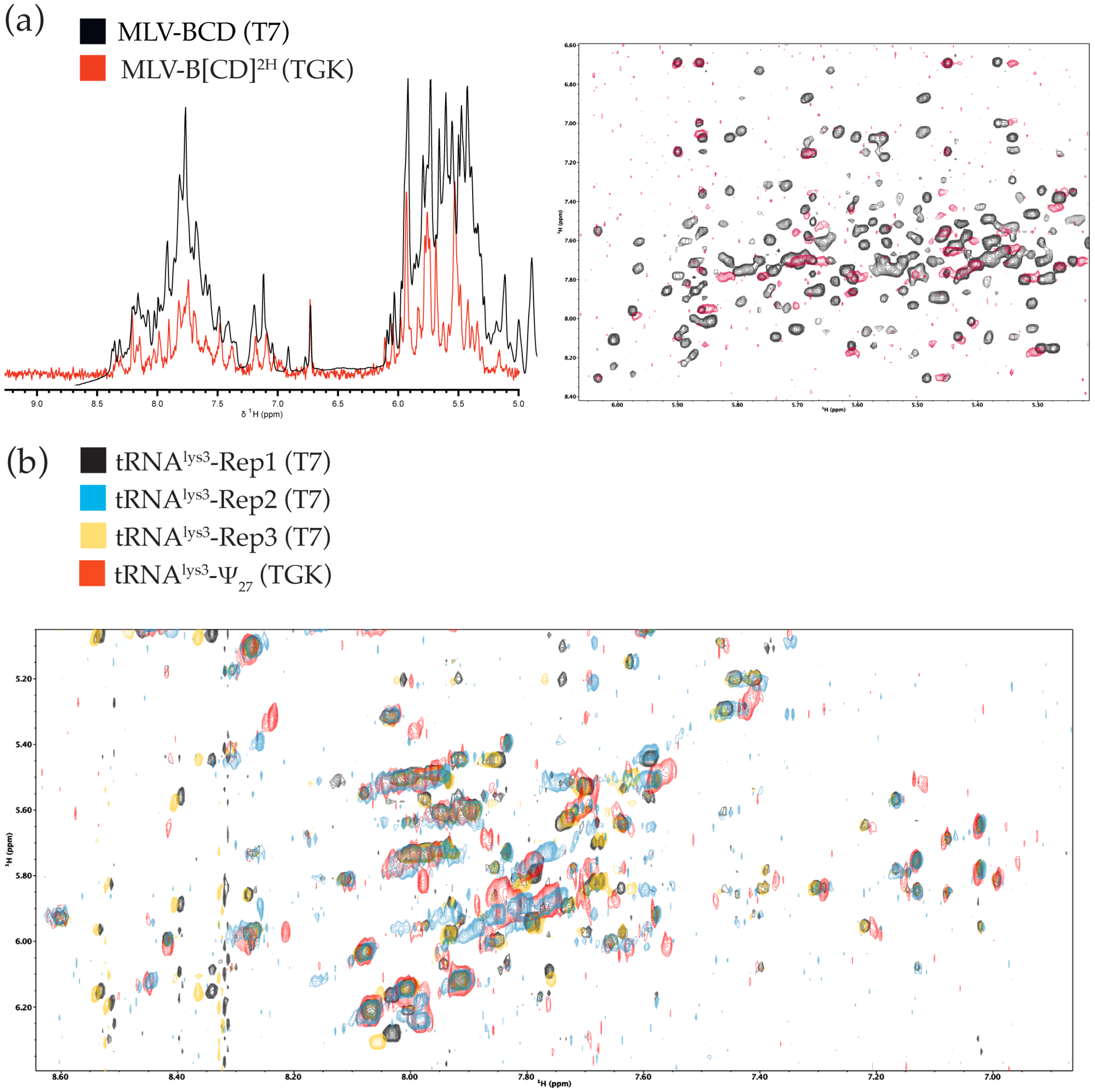

Supplementary Figure 2: NMR data of MLV-BCD and tRNA samples
(a) 1D (left) and 2D (right) overlays of MLV-BCD made by T7 (black) and MLV-B[CD]^{2H} made by SegModTeX (red) **(b)** 2D overlays of tRNA^{Lys3} replicates made by T7 (black, blue, and yellow) showing changes in chemical shifts sensitive to solution conditions. tRNA^{Lys3}-Ψ made by SegModTeX (red) demonstrates chemical shifts that mostly matches replicate 2 except for regions surrounding Ψ incorporation.

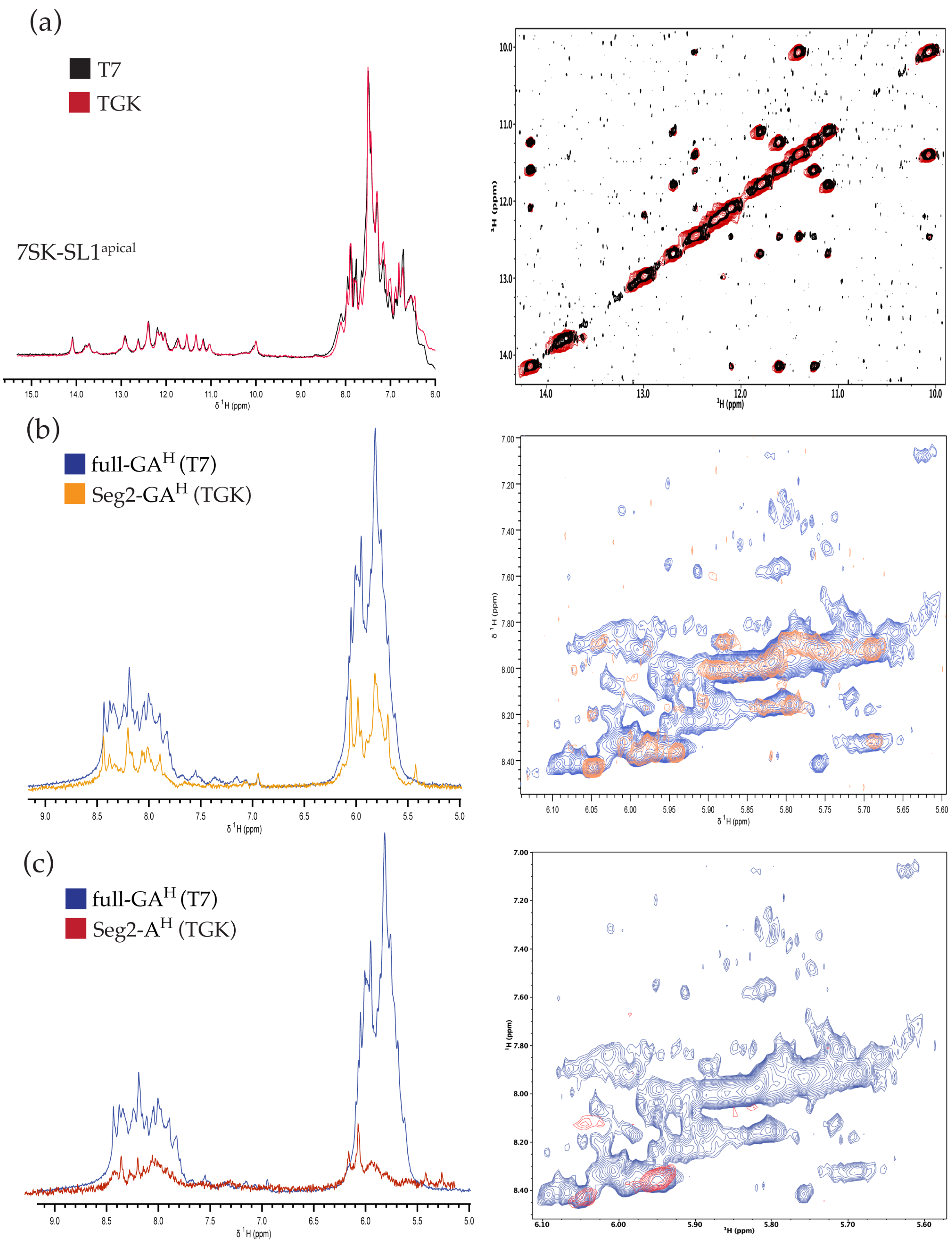

Supplementary Figure 3: NMR data of 7SK snRNA samples
(a-c) 1D (left) and 2D (right) overlays of 7SK transcripts made by T7 [black in (a), blue in (b) and (c)] and by SegModTeX [red in (a) and (c), orange in (b)]. For labeling schema, see Fig. 1 for (a) and Fig. 4 for (b) and (c) in main text.

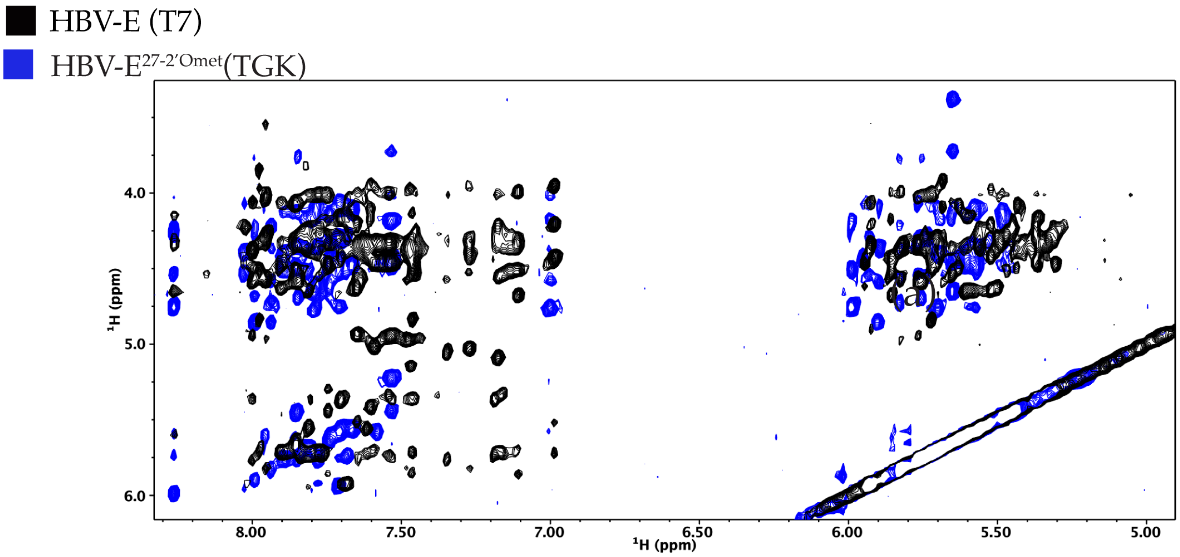

Supplementary Figure 4: NMR data of 2′-OMe HBV-ε

2D overlay of T7 (black) and SegModTex (blue) synthesized HBV-ε samples. The incorporation of 2′-OMe is characterized by the upfield-shifted methyl resonance only in the SegModTex sample although as noted in Fig. 6, this results in a stalled extension.

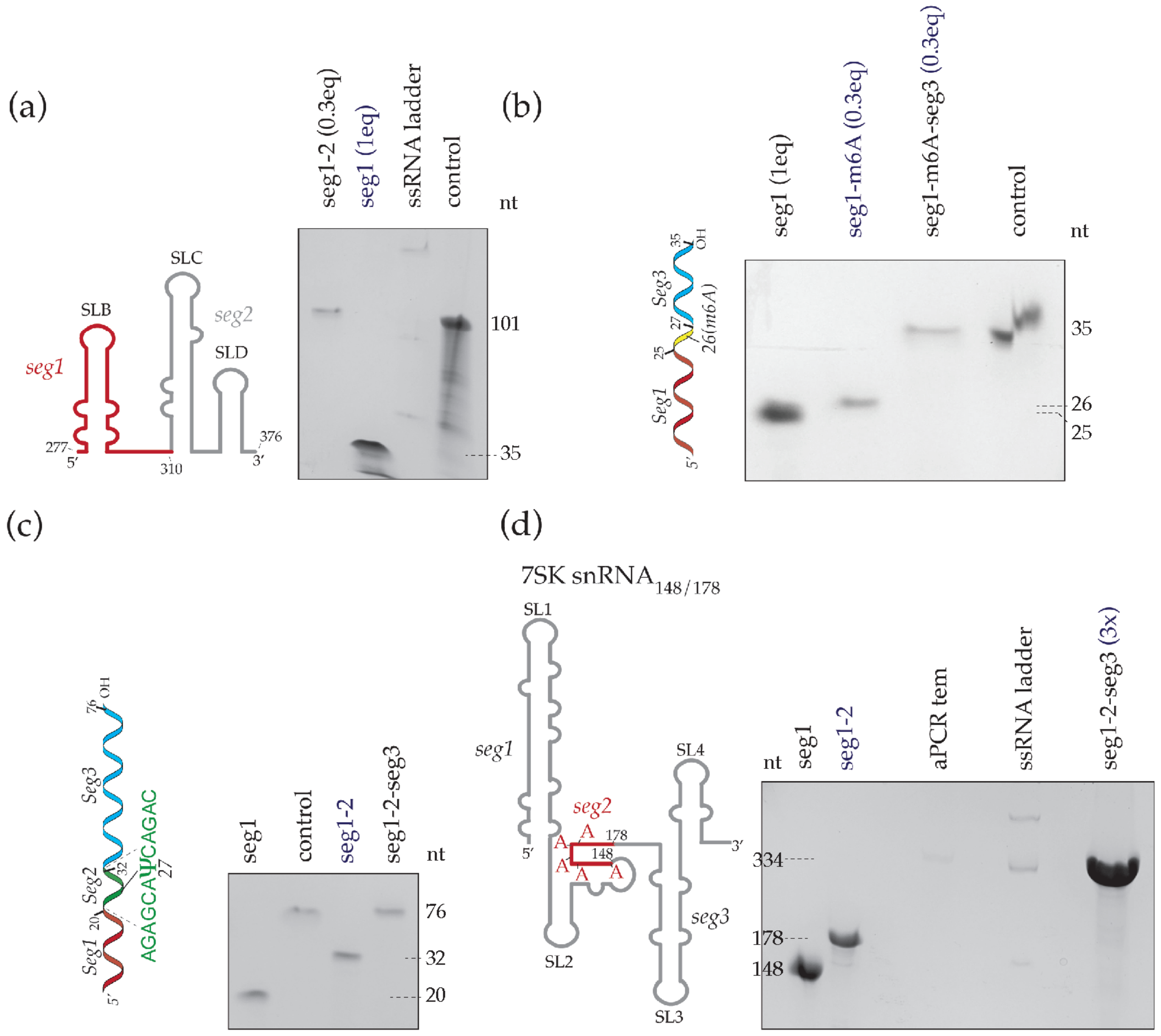

Supplementary Figure 5: Replicates of SegModTeX reaction
Schematics (left) and PAGEs (right) for replicates of MLV-BCD **(a)**, HBV-ε **(b)**, tRNA^{lys3} (c), and 7SK snRNA (d), presented in Figs. 3-5 of the main text.

Construct	Guanidine washes	Gel purification
HBV-ε, n=3	99.27 % s^2 = 3.15	-
tRNA-Lys, n=3	97.55% s^2 = 2.08	-
MLV-B[CD]^{2H} 1 purification step	-	76%
HBV-ε -2^{19}F-26A 1 purification step	-	84%
7SK snRNA$_{180/253}$ 2 purification steps	-	54%

Supplementary Table 1: Yield quantifications

To assess yields, data on representative samples are presented after six washes with 5M Guanidinium-HCl to flush out unincorporated rNTPs and digested dNTPs **(column 2)** or after polyacrylamide gel electrophoresis and subsequent electroelution **(column 3)**.
